# (*E*)-1,5-Di­phenyl­pent-2-en-4-yn-1-one

**DOI:** 10.1107/S1600536813013044

**Published:** 2013-05-18

**Authors:** Alexander S. Bunev, Vladimir E. Statsyuk, Nina V. Utekhina, Victor N. Khrustalev

**Affiliations:** a Department of Chemistry and Chemical Technology, Togliatti State University, 14 Belorusskaya St, Togliatti 445667, Russian Federation; b X-Ray Structural Centre, A.N. Nesmeyanov Institute of Organoelement Compounds, Russian Academy of Sciences, 28 Vavilov St, B-334, Moscow 119991, Russian Federation

## Abstract

The title compound, C_17_H_12_O, has an *E* conformation about the C=C bond. The C—C C—C torsion angle is 7.7 (2)°, and the mean planes of the phenyl­ethyl­enone [r.m.s. deviation = 0.059 (1) Å] and phenyl­acetyl­ene [r.m.s. deviation = 0.023 (1) Å] fragments form a dihedral angle of 14.16 (7)°. In the crystal, weak C—H⋯O inter­actions link the mol­ecules into zigzag chains propagated in [010].

## Related literature

For the synthesis and properties of enynones, see: Toshima *et al.* (1999 ▶); Ohe *et al.* (2002 ▶); Miki *et al.* (2002 ▶); Kuroda *et al.* (2004 ▶); Casey & Strotman (2005 ▶). For the crystal structures of related compounds, see: König *et al.* (1995 ▶); Chen & Liu (2008 ▶); Lu *et al.* (2009 ▶).
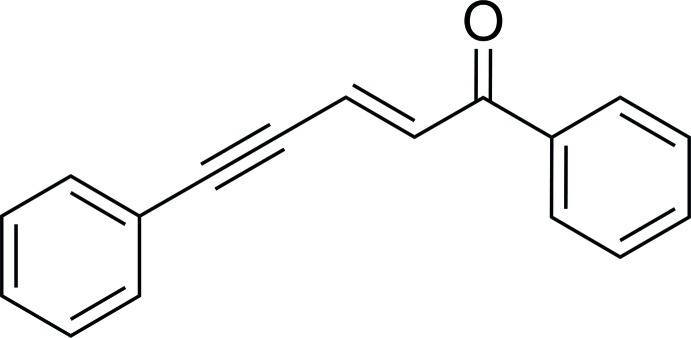


## Experimental

### 

#### Crystal data

C_17_H_12_O
*M* *_r_* = 232.27
Orthorhombic, 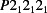

*a* = 5.4696 (4) Å
*b* = 13.7164 (10) Å
*c* = 16.3117 (11) Å
*V* = 1223.76 (15) Å^3^
*Z* = 4
Mo *K*α radiation
μ = 0.08 mm^−1^
*T* = 120 K
0.30 × 0.25 × 0.20 mm

#### Data collection

Bruker APEXII CCD diffractometer
Absorption correction: multi-scan (*SADABS*; Sheldrick, 2003 ▶) *T*_min_ = 0.977, *T*_max_ = 0.985
15765 measured reflections
3576 independent reflections
2987 reflections with *I* > 2σ(*I*)
*R*_int_ = 0.058

#### Refinement

*R*[*F* ^2^ > 2σ(*F* ^2^)] = 0.057
*wR*(*F* ^2^) = 0.107
*S* = 1.07
3576 reflections
163 parameters
H-atom parameters constrained
Δρ_max_ = 0.20 e Å^−3^
Δρ_min_ = −0.28 e Å^−3^

### 

Data collection: *APEX2* (Bruker, 2005 ▶); cell refinement: *SAINT* (Bruker, 2001 ▶); data reduction: *SAINT*; program(s) used to solve structure: *SHELXTL* (Sheldrick, 2008 ▶); program(s) used to refine structure: *SHELXTL*; molecular graphics: *SHELXTL*; software used to prepare material for publication: *SHELXTL*.

## Supplementary Material

Crystal structure: contains datablock(s) global, I. DOI: 10.1107/S1600536813013044/cv5408sup1.cif

Structure factors: contains datablock(s) I. DOI: 10.1107/S1600536813013044/cv5408Isup2.hkl

Supplementary material file. DOI: 10.1107/S1600536813013044/cv5408Isup3.cml

Additional supplementary materials:  crystallographic information; 3D view; checkCIF report

## Figures and Tables

**Table 1:** Hydrogen-bond geometry (Å, °)

*D*—H⋯*A*	*D*—H	H⋯*A*	*D*⋯*A*	*D*—H⋯*A*
C16—H16⋯O1^i^	0.95	2.54	3.165 (2)	124
C17—H17⋯O1^i^	0.95	2.61	3.202 (2)	121

Symmetry code: (i) 
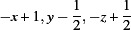
.

**Fig. 1.**
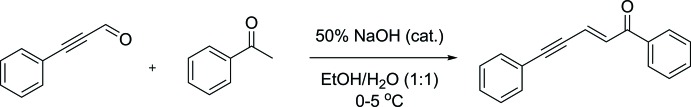
The condensation reaction of 3-phenylpropiolaldehyde with acetophenone.

**Fig. 2.**
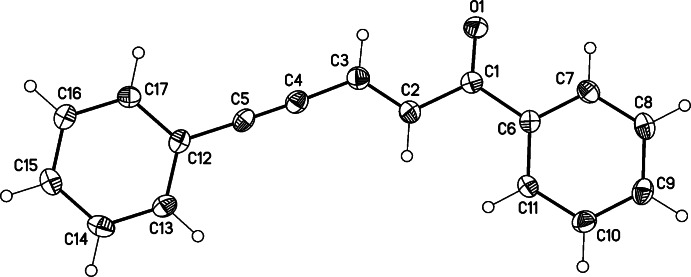
Molecular structure of I. Displacement ellipsoids are shown at the 50% probability level. H atoms are presented as small spheres of arbitrary radius.

**Fig. 3.**
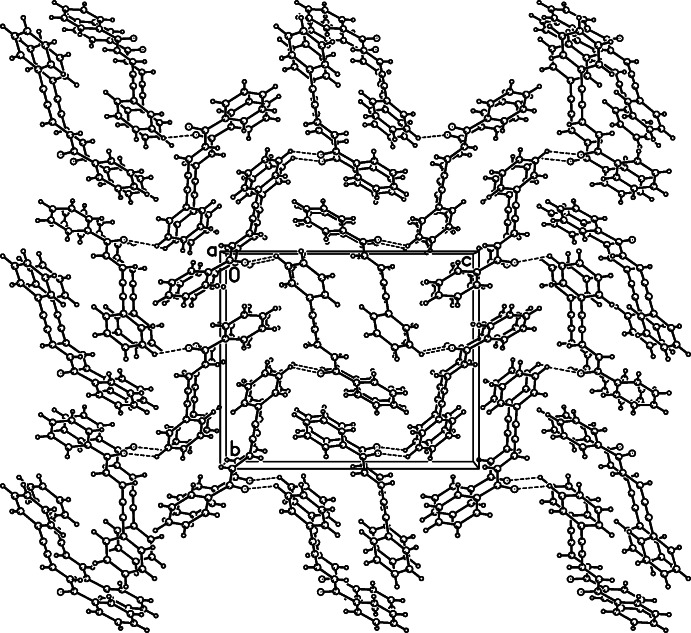
A portion of the crystal structure demonstrating the H-bonded zigzag chains of I along the b axis. The weak intermolecular C—H···O hydrogen bonds are depicted by dashed lines.

## supplementary crystallographic information

### Comment

*cis*- and *trans*-Enynones are important building blocks in organic synthesis, particularly, in total synthesis of natural products (Toshima *et al.*, 1999), in reactions of conjugated addition (Kuroda *et al.*, 2004; Casey & Strotman, 2005), in metal catalyzed furan formations *via* 2-furyl carbene complexes (Miki *et al.*, 2002), and as substrate precursors in [3,3]-sigmatropic rearrangement (Ohe *et al.*, 2002). In this work, we present the title compound, I, prepared by the condensation reaction of 3-phenylpropiolaldehyde with acetophenone at reduced temperature (0–5°C) (Fig. 1).

Compound I represents the *E*-isomer about the C2═C3 bond and adopts almost planar structure (Figure 2). The small folding of 14.16 (7)° is between the mean planes of the phenylethylenone (O1/C1/C2/C3/C6/C7/C8/C9/C10/C11, r.m.s. deviation = 0.059 (1) Å) and phenylacetylene (C3/C4/C5/C12/C13/C14/C15/C16/C17, r.m.s. deviation = 0.023 (1) Å) fragments. The C3—C4≡C5—C12 torsion angle is 7.7 (2)°, and the bond elongations observed within the C3—C4≡C5—C12 acetylene fragment (C3—C4 1.419 (3) Å, C5—C12 1.432 (2) Å, C4≡C5 1.201 (3) Å) are characteristic for compounds of this type (König *et al.*, 1995; Chen & Liu, 2008; Lu *et al.*, 2009).

In the crystal, the molecules of I form *zigzag* chains along the *b* axis by the weak intermolecular C—H···O hydrogen bonds (Table 1). The crystal packing of the chains is stacking along the *a* axis (Figure 3).

### Experimental

A solution of sodium hydroxide (0.24 g, 6 mmol) in H_2_O (1 ml) was added dropwise over 15 min to a mixture of 3-phenylpropiolaldehyde (1.0 g, 8 mmol) and acetophenone (0.9 ml, 0.93 g, 8 mmol) in 50% EtOH (10 ml) cooled to 0°C. During the reaction, the temperature was not allowed to exceed 5 °C. The mixture was stirred for 10 h. The precipitated solid was filtered and crystallized from EtOH. Yield is 86%. The single-crystal of the product was obtained by slow crystallization from methanol. *M*.p. = 372–373 K. IR (KBr), ν/cm^-1^: 3063, 2191, 1661, 1597, 1580, 1337, 1308, 1254, 1211. ^1^H NMR (400 MHz, CDCl_3_, 303 K): δ = 7.13 (d, 1H, J = 15.6), 7.33–7.37 (m, 3H), 7.43 (d, 1H, J = 15.2), 7.47–7.53 (m, 4H), 7.55–7.57 (m, 1H), 7.96–7.99 (m, 2H). ^13^C NMR (100 MHz, CDCl_3_, 303 K): δ = 87.7, 99.3, 122.2, 125.1, 128.5, 128.5, 128.7, 129.4, 132.0, 133.0, 137.2, 133.2, 188.8. Anal. Calcd. for C_17_H_12_O: C, 87.90; H, 5.21. Found: C, 87.78; H, 5.29.

### Refinement

All hydrogen atoms were placed in the calculated positions with C—H = 0.95 Å and refined in the riding model with fixed isotropic displacement parameters [*U*_iso_(H) = 1.2*U*_eq_(C)].

### Crystal data

**Table:**

C_17_H_12_O	*F*(000) = 488
*M**_r_* = 232.27	*D*_x_ = 1.261 Mg m^−^^3^
Orthorhombic, *P*2_1_2_1_2_1_	Mo *K*α radiation, λ = 0.71073 Å
Hall symbol: P 2ac 2ab	Cell parameters from 2940 reflections
*a* = 5.4696 (4) Å	θ = 2.5–31.0°
*b* = 13.7164 (10) Å	µ = 0.08 mm^−^^1^
*c* = 16.3117 (11) Å	*T* = 120 K
*V* = 1223.76 (15) Å^3^	Prism, yellow
*Z* = 4	0.30 × 0.25 × 0.20 mm

### Data collection

**Table:**

Bruker APEXII CCD diffractometer	3576 independent reflections
Radiation source: fine-focus sealed tube	2987 reflections with *I* > 2σ(*I*)
Graphite monochromator	*R*_int_ = 0.058
φ and ω scans	θ_max_ = 30.0°, θ_min_ = 1.9°
Absorption correction: multi-scan (*SADABS*; Sheldrick, 2003)	*h* = −7→7
*T*_min_ = 0.977, *T*_max_ = 0.985	*k* = −19→19
15765 measured reflections	*l* = −22→22

### Refinement

**Table:**

Refinement on *F*^2^	Primary atom site location: structure-invariant direct methods
Least-squares matrix: full	Secondary atom site location: difference Fourier map
*R*[*F*^2^ > 2σ(*F*^2^)] = 0.057	Hydrogen site location: inferred from neighbouring sites
*wR*(*F*^2^) = 0.107	H-atom parameters constrained
*S* = 1.07	*w* = 1/[σ^2^(*F*_o_^2^) + (0.0338*P*)^2^ + 0.3124*P*] where *P* = (*F*_o_^2^ + 2*F*_c_^2^)/3
3576 reflections	(Δ/σ)_max_ < 0.001
163 parameters	Δρ_max_ = 0.20 e Å^−^^3^
0 restraints	Δρ_min_ = −0.28 e Å^−^^3^

### Special details

**Table:**

Geometry. All e.s.d.'s (except the e.s.d. in the dihedral angle between two l.s. planes) are estimated using the full covariance matrix. The cell e.s.d.'s are taken into account individually in the estimation of e.s.d.'s in distances, angles and torsion angles; correlations between e.s.d.'s in cell parameters are only used when they are defined by crystal symmetry. An approximate (isotropic) treatment of cell e.s.d.'s is used for estimating e.s.d.'s involving l.s. planes.
Refinement. Refinement of *F*^2^ against ALL reflections. The weighted *R*-factor *wR* and goodness of fit *S* are based on *F*^2^, conventional *R*-factors *R* are based on *F*, with *F* set to zero for negative *F*^2^. The threshold expression of *F*^2^ > σ(*F*^2^) is used only for calculating *R*-factors(gt) *etc*. and is not relevant to the choice of reflections for refinement. *R*-factors based on *F*^2^ are statistically about twice as large as those based on *F*, and *R*- factors based on ALL data will be even larger.

### Fractional atomic coordinates and isotropic or equivalent isotropic displacement parameters (Å^2^)

**Table:**

	*x*	*y*	*z*	*U*_iso_*/*U*_eq_	
O1	0.1245 (3)	0.55925 (10)	0.38879 (8)	0.0335 (4)	
C1	0.2582 (3)	0.54777 (13)	0.44784 (10)	0.0206 (4)	
C2	0.4701 (4)	0.48088 (14)	0.44289 (11)	0.0256 (4)	
H2	0.5833	0.4784	0.4870	0.031*	
C3	0.5045 (3)	0.42448 (13)	0.37800 (11)	0.0244 (4)	
H3	0.3930	0.4310	0.3337	0.029*	
C4	0.6951 (3)	0.35500 (13)	0.36950 (11)	0.0236 (4)	
C5	0.8456 (3)	0.29185 (13)	0.36230 (10)	0.0220 (4)	
C6	0.2084 (3)	0.60079 (12)	0.52645 (11)	0.0186 (3)	
C7	−0.0018 (3)	0.65863 (13)	0.53149 (11)	0.0229 (4)	
H7	−0.1086	0.6634	0.4857	0.027*	
C8	−0.0548 (3)	0.70902 (13)	0.60295 (11)	0.0243 (4)	
H8	−0.1989	0.7474	0.6062	0.029*	
C9	0.1022 (3)	0.70358 (13)	0.66981 (12)	0.0252 (4)	
H9	0.0661	0.7386	0.7186	0.030*	
C10	0.3109 (4)	0.64699 (14)	0.66512 (12)	0.0259 (4)	
H10	0.4186	0.6434	0.7107	0.031*	
C11	0.3639 (3)	0.59521 (13)	0.59370 (11)	0.0210 (4)	
H11	0.5069	0.5560	0.5910	0.025*	
C12	1.0198 (3)	0.21457 (12)	0.35339 (10)	0.0198 (4)	
C13	1.2102 (4)	0.20245 (14)	0.40995 (11)	0.0239 (4)	
H13	1.2228	0.2451	0.4557	0.029*	
C14	1.3797 (4)	0.12875 (13)	0.39951 (12)	0.0265 (4)	
H14	1.5079	0.1207	0.4382	0.032*	
C15	1.3632 (4)	0.06627 (13)	0.33257 (12)	0.0254 (4)	
H15	1.4813	0.0162	0.3253	0.031*	
C16	1.1752 (3)	0.07688 (13)	0.27652 (11)	0.0240 (4)	
H16	1.1632	0.0337	0.2312	0.029*	
C17	1.0046 (3)	0.15044 (13)	0.28663 (10)	0.0213 (4)	
H17	0.8760	0.1576	0.2480	0.026*	

### Atomic displacement parameters (Å^2^)

**Table:**

	*U*^11^	*U*^22^	*U*^33^	*U*^12^	*U*^13^	*U*^23^
O1	0.0380 (8)	0.0373 (8)	0.0251 (7)	0.0140 (7)	−0.0102 (6)	−0.0036 (6)
C1	0.0223 (9)	0.0184 (8)	0.0211 (8)	−0.0014 (7)	0.0001 (7)	0.0016 (7)
C2	0.0276 (10)	0.0249 (9)	0.0242 (9)	0.0061 (8)	−0.0026 (8)	−0.0011 (8)
C3	0.0264 (9)	0.0247 (9)	0.0222 (9)	0.0025 (8)	0.0001 (8)	0.0028 (7)
C4	0.0277 (9)	0.0242 (9)	0.0190 (8)	−0.0007 (8)	0.0016 (8)	0.0004 (7)
C5	0.0261 (9)	0.0226 (8)	0.0174 (8)	−0.0030 (8)	0.0037 (7)	−0.0017 (7)
C6	0.0194 (8)	0.0154 (8)	0.0211 (8)	−0.0025 (7)	0.0028 (7)	0.0020 (6)
C7	0.0219 (9)	0.0209 (9)	0.0258 (9)	0.0018 (8)	−0.0007 (8)	0.0035 (7)
C8	0.0215 (9)	0.0192 (9)	0.0323 (10)	0.0016 (7)	0.0049 (8)	0.0005 (8)
C9	0.0268 (10)	0.0209 (9)	0.0278 (9)	−0.0029 (8)	0.0050 (8)	−0.0056 (8)
C10	0.0253 (9)	0.0286 (9)	0.0237 (9)	−0.0024 (8)	−0.0024 (8)	−0.0033 (8)
C11	0.0178 (8)	0.0208 (8)	0.0246 (9)	0.0013 (7)	0.0005 (7)	0.0001 (7)
C12	0.0235 (9)	0.0186 (8)	0.0172 (8)	−0.0030 (7)	0.0055 (7)	0.0013 (6)
C13	0.0280 (9)	0.0243 (9)	0.0194 (8)	−0.0031 (8)	−0.0001 (7)	0.0000 (7)
C14	0.0227 (9)	0.0268 (10)	0.0300 (10)	−0.0027 (8)	−0.0068 (8)	0.0058 (8)
C15	0.0221 (9)	0.0223 (9)	0.0319 (10)	0.0031 (8)	0.0027 (8)	0.0020 (8)
C16	0.0276 (10)	0.0214 (9)	0.0230 (9)	−0.0012 (8)	0.0031 (8)	−0.0018 (7)
C17	0.0218 (9)	0.0229 (9)	0.0193 (8)	−0.0015 (8)	−0.0002 (7)	0.0021 (7)

### Geometric parameters (Å, º)

**Table:**

O1—C1	1.220 (2)	C9—H9	0.9500
C1—C2	1.480 (3)	C10—C11	1.395 (2)
C1—C6	1.499 (2)	C10—H10	0.9500
C2—C3	1.324 (3)	C11—H11	0.9500
C2—H2	0.9500	C12—C13	1.401 (2)
C3—C4	1.419 (3)	C12—C17	1.402 (2)
C3—H3	0.9500	C13—C14	1.382 (3)
C4—C5	1.201 (3)	C13—H13	0.9500
C5—C12	1.432 (2)	C14—C15	1.391 (3)
C6—C11	1.390 (2)	C14—H14	0.9500
C6—C7	1.399 (2)	C15—C16	1.384 (3)
C7—C8	1.386 (2)	C15—H15	0.9500
C7—H7	0.9500	C16—C17	1.384 (3)
C8—C9	1.390 (3)	C16—H16	0.9500
C8—H8	0.9500	C17—H17	0.9500
C9—C10	1.383 (3)		
			
O1—C1—C2	120.42 (16)	C9—C10—H10	119.9
O1—C1—C6	120.26 (16)	C11—C10—H10	119.9
C2—C1—C6	119.33 (16)	C6—C11—C10	120.25 (17)
C3—C2—C1	121.14 (17)	C6—C11—H11	119.9
C3—C2—H2	119.4	C10—C11—H11	119.9
C1—C2—H2	119.4	C13—C12—C17	118.73 (17)
C2—C3—C4	125.09 (18)	C13—C12—C5	121.01 (16)
C2—C3—H3	117.5	C17—C12—C5	120.25 (16)
C4—C3—H3	117.5	C14—C13—C12	120.28 (17)
C5—C4—C3	176.0 (2)	C14—C13—H13	119.9
C4—C5—C12	178.4 (2)	C12—C13—H13	119.9
C11—C6—C7	119.19 (16)	C13—C14—C15	120.25 (17)
C11—C6—C1	122.47 (16)	C13—C14—H14	119.9
C7—C6—C1	118.34 (16)	C15—C14—H14	119.9
C8—C7—C6	120.27 (17)	C16—C15—C14	120.14 (18)
C8—C7—H7	119.9	C16—C15—H15	119.9
C6—C7—H7	119.9	C14—C15—H15	119.9
C7—C8—C9	120.27 (17)	C15—C16—C17	119.92 (17)
C7—C8—H8	119.9	C15—C16—H16	120.0
C9—C8—H8	119.9	C17—C16—H16	120.0
C10—C9—C8	119.78 (17)	C16—C17—C12	120.67 (17)
C10—C9—H9	120.1	C16—C17—H17	119.7
C8—C9—H9	120.1	C12—C17—H17	119.7
C9—C10—C11	120.24 (18)		
			
O1—C1—C2—C3	−7.3 (3)	C8—C9—C10—C11	−0.2 (3)
C6—C1—C2—C3	172.28 (18)	C7—C6—C11—C10	−0.2 (3)
C1—C2—C3—C4	−176.86 (17)	C1—C6—C11—C10	179.16 (17)
O1—C1—C6—C11	−175.68 (18)	C9—C10—C11—C6	0.6 (3)
C2—C1—C6—C11	4.8 (3)	C17—C12—C13—C14	0.3 (3)
O1—C1—C6—C7	3.7 (3)	C5—C12—C13—C14	−178.64 (17)
C2—C1—C6—C7	−175.85 (16)	C12—C13—C14—C15	0.3 (3)
C3—C4—C5—C12	7.7 (2)	C13—C14—C15—C16	−0.7 (3)
C11—C6—C7—C8	−0.5 (3)	C14—C15—C16—C17	0.7 (3)
C1—C6—C7—C8	−179.91 (17)	C15—C16—C17—C12	−0.1 (3)
C6—C7—C8—C9	0.9 (3)	C13—C12—C17—C16	−0.3 (3)
C7—C8—C9—C10	−0.5 (3)	C5—C12—C17—C16	178.58 (16)

### Hydrogen-bond geometry (Å, º)

**Table:**

*D*—H···*A*	*D*—H	H···*A*	*D*···*A*	*D*—H···*A*
C16—H16···O1^i^	0.95	2.54	3.165 (2)	124
C17—H17···O1^i^	0.95	2.61	3.202 (2)	121

Symmetry code: (i) −*x*+1, *y*−1/2, −*z*+1/2.
